# Effects of Maternal Decisional Authority and Media Use on Vaccination for Children in Asian Countries

**DOI:** 10.3390/medicina54060105

**Published:** 2018-12-07

**Authors:** Minsung Sohn, Leesa Lin, Minsoo Jung

**Affiliations:** 1 BK21Plus Program in Embodiment: Health-Society Interaction, Department of Public Health Sciences, Graduate School of Korea University, Seoul 02841, Korea; minsinge@naver.com; 2 Department of Public Health, Environments and Society, London School of Hygiene & Tropical Medicine, London WC1H 9SH, UK; leesa_lin@hms.harvard.edu; 3 Department of Global Health and Social Medicine, Harvard Medical School, Boston 02115, MA, USA; 4 Department of Health Science, Dongduk Women’s University, Seoul 136-714, Korea; 5 Center for Community-Based Research, Dana-Farber/Harvard Cancer Center, Boston 02215, MA, USA

**Keywords:** vaccination, women’s decisional authority, media use, health communication, Southeast Asia, South Asia

## Abstract

*Background and objectives:* It is now accepted that vaccination is a critical public health strategy in preventing child morbidity and mortality. Understanding factors that promote vaccination is a critical first step. The objective of this study was to investigated associations of maternal decisional authority and media use on vaccination for children in six South and Southeast Asian countries. *Materials and Methods:* Data come from demographic and health surveys conducted in Bangladesh, Cambodia, Indonesia, Nepal, Pakistan, and the Philippines between 2011 and 2014 (N = 45,168 women). Main outcome variables were four types of basic vaccination for children. Independent variables were maternal decisional authority and media use. Hierarchical multivariable regression analyses were performed to examine associations. *Results:* Children of mothers who had more decisional authority were more likely to be vaccinated compared to those participants who did not have such authority. The likelihood to have their children vaccinated was higher among women who frequently used media than those who did not use media. *Conclusions:* Maternal decisional authority and media use are related to improved vaccination for children. To increase vaccination rates in developing countries in South and Southeast Asia, programs and policies that promote maternal decisional authority and the use of media for health need to be implemented to help families and local communities.

## 1. Introduction

The development of mass media has enabled women to participate in daily affairs outside home, including education, health, governance, and other areas [1]. Empowerment of women is a process of providing power to woman to become free from the control of others, that is, to assume power to control their own lives and determine their own conditions [2,3]. Women’s empowerment may also be understood as a process of providing equal rights, opportunities, responsibilities, and power positions to women so that they can play a role in society on par with men [4,5].

Inequality in maternal and child healthcare utilization is related to women’s economic, educational, and empowerment statuses (3Es) [6]. These 3Es are indispensable for realizing the Millennium Development Goals (MDGs). Undesirable social environment of insufficient 3Es can weaken the effect of public health interventions and consolidate inequality [7]. In developing countries, a diverse set of delays (e.g., delays in obtaining medical services, delays in arriving at medical institutions, and delays in providing medical services) can adversely affect maternal and child health [8]. Delays related to health and medical care are potential causes of high child mortality ratios (CMRs). These 3Es have been found to be one set of important antecedents to CMR [6,7,8].

Today, information technology has changed the communication paradigm between medical professionals and the public, making it no longer difficult to reach a large number of people. Widespread availability of information through the penetration of mass media has played a significant role in the empowerment of women by easing access and reducing barriers [1,2,3]. Despite its importance for maternal and child health, female empowerment in developing countries has not been adequately addressed yet. Health-related empowerment of women here refers to their ability to make their own decisions, including vaccinations for their children [9]. More appropriately, it may be defined as the decisional authority of women of childbearing age over maternal health. It also encompasses safe home and public spaces for women, ensuring their economic autonomy and security, and increasing their participation and decision-making power. For example, violence against women and girls is rooted in gender-based discrimination, social norms, and gender stereotypes that perpetuate such violence [6,9].

For countries in South and Southeast Asia, the lack of women’s decision-making and the lack of economic empowerment can cause worsening of health for women and children [3,4,6,8]. Especially, Pakistan is still suffering from the outbreak of polio. There were also unusual cases that workers who had polio were killed. Empowering women in these developing countries has become a primary policy goal since the World Congress on Women, 1995 held in Beijing, China [9]. Although, vaccines are one of the most cost-effective public health measures available, they are undervalued throughout the world. Children in Southeast Asia are still suffering from various vaccine-preventable diseases. Protection against newly developed vaccines such as pneumococcus, rotavirus, and human papillomavirus infections were inadequate in most of these countries in South and Southeast Asia [10]. Promoting coverage of newly developed vaccines will benefit a great number of children in this area. The purpose of this study was to determine the effect of women’s decisional authority and media use on decisions related to vaccinations in South and Southeast Asian countries (Bangladesh, Cambodia, Indonesia, Nepal, Pakistan, and the Philippines).

## 2. Methods

### 2.1. Study Sample

Data used for this study were pooled from the latest Demographic and Health Surveys (DHS) conducted in six available South and Southeast Asia countries (Bangladesh: 2011; Cambodia: 2014; Indonesia: 2012; Nepal: 2011; Pakistan: 2012; the Philippines: 2013) with different survey years due to country-specific situations. Nationally representative surveys of 5000 to 30,000 households were periodically performed in low- and middle-income countries. DHS were based on multi-stage stratified sample of households from census enumeration areas in urban or rural villages. Each country’s sample size was calculated by applying that country’s urban and rural population proportions and the country’s gender ratio. In all households, women aged 13–49 years were eligible to participate. Country-specific details of the survey can be found on the following web page [11]. DHS data including demographic characteristics, women’s decisional authority, mass media use, and vaccination status were obtained through face-to-face interviews. Response rates were higher than 90% among all DHS participants in South and Southeast Asia countries. After excluding responses with missing values, a total of 45,168 women in Southeast Asia countries were used in the final analysis.

### 2.2. Study Design

This was a cross-sectional study to examine the association between women’s decisional authority, mass media and vaccination for children (i.e., vaccine coverage rates of the children) using DHS data obtained from South and Southeast Asia.

### 2.3. Measures

#### 2.3.1. Dependent Variables

The dependent variable for this study was vaccination for children. It was assessed by asking “Now think about getting the vaccine for your child/children. Have you vaccinated one or all of your children?” The response was categorized as “yes” or “no”. Four binary variables were generated for the following four vaccinations: Bacillus Calmette-Guerin (BCG), diphtheria + pertussis + tetanus (DPT), poliomyelitis (polio), and measles.

#### 2.3.2. Independent Variables

For this study, women’s decisional authority and media were used as independent variables.

Women’s decisional authority was considered as women having two dimensions of decision-making in daily life and attitudes toward intimate partner violence in DHS data. Decision making in daily life was measured using the following three questions: “Who usually makes decisions about health care for yourself?”; “Who usually makes decisions about making major household purchases?”; and “Who usually makes decisions about visits to your family or relatives?” Responses were grouped into three categories: (1) partner decides, (2) partner and woman decide jointly, and (3) woman decides. Regarding attitudes toward partner violence, the following five domestic scenarios with yes-no questions was asked: “In your opinion, is a husband justified in hitting or beating his wife in the following situations? (1) goes out without telling him, (2) neglects children, (3) argues with him, (4) refuses to have sex, and (5) burns food.”

Exploratory factor analysis was conducted with a total of nine total questions to multi-dimensionally assess women’s decisional authority [11]. Exploratory factor analysis was performed with Varimax rotation to determine the construct validity. Factors used to construct the women’s decisional authority index had eigenvalues greater than 1 and factor loadings greater than 0.40. The first factor, named decision-making in daily life, accounted for 24.7% of the total variance (Cronbach’s alpha = 0.733). The use of this dimension was validated in previous studies on women empowerment [11]. This factor included three of nine decisional authorities: “decisions about health care for yourself; decisions about making major household purchases; and decisions about visits to your family or relatives” In accordance with approaches used in previous studies [12], a second factor, named freedom from domination, explained 24.1% of the total variance (Cronbach’s alpha = 0.816). This factor included two potential decisional authorities: “goes out without telling him; and neglects children.” A third factor, named self-assertion, explained 23.2% of the total variance (Cronbach’s alpha = 0.763). This factor included the remaining three potential decisional authority dimensions: “argues with her husband; refuses to have sex; and burns food.” These factors were transformed into scores by aggregating answers of relevant sub-questions.

Media use was assessed in this study to identify other effects on outcome variables. How often participants used three mass media types (newspaper, radio, and television) was considered. Responses were grouped into four categories: (1) not at all, (2) less than once a week, (3) at least once a week, and (4) almost every day.

#### 2.3.3. Covariates

It has been previously reported that child and maternal health are related to demographic characteristics of respondents [13,14,15]. Accordingly, mother’s age, socio-economic position (SEP; i.e., educational attainment, household income), and location were considered as covariates in this study. Participants were grouped into the following age categories: 13–19, 20–24, 25–29, 30–34, 35–39, 40–44, and 45–49 years old. Educational attainments were grouped into the following categories: no schooling, elementary school/associate degree, middle school/associate degree, and high school/associate degree or higher. Household income was grouped into the following standard categories: the lowest 20% (first quintile), low 20%, middle 20%, high 20%, and the highest 20% (fifth quintile). Location was grouped into urban and rural.

### 2.4. Statistical Analyses

First, general characteristics of the sample were described. Distribution of vaccination of children and utilization of maternal health service were also assessed. Second, hierarchical multivariable logistic regression analysis was performed to examine the association between women’s decisional authority factors and vaccination of children after conducting exploratory factor analysis. Poisson regression was used because dependent variables were count measures with a skewed nonparametric distribution that standard parametric approaches such as linear regression were not statistically appropriate [16]. The association between women’s decisional authority and vaccination for her children (mother’s vaccine coverage rates for the children and utilization of maternal health service) stratified by frequency of media use (newspaper, radio, and television) was also assessed. All statistical analyses were conducted using STATA v. 14.0 (STATA, College Station, TX, USA).

### 2.5. Ethics Statement

Approval for the study was granted by the Korea National Institute for Bioethics Policy Institutional Review Board (25 November 2016; P01-201611-21-009). All participants gave written informed consent to participate. The Ethics Committees of the Demographic Health Survey approved this consent procedure. During the investigation process, no information that could distinguish individual participants was collected.

## 3. Results

### 3.1. Sample Demographic Characteristics

As detailed in Table 1, a total of 45,168 female participants were recruited from six countries, including 15,579 from Indonesia (34.5%), 7009 from Pakistan (15.5%), 6917 from Bangladesh (15.3%), 6169 from Cambodia (13.7%), 6125 from the Philippines (13.6%), and 3369 from Nepal (7.5%). Age patterns were similar across countries among participants. Those who were 25–29 years old accounted for the most, ranging from 25.26% in Philippines to 35.44% in Nepal. Regarding educational attainment, most participants had middle school/associate degree in Indonesia (53.64%), the Philippines (48.83%), and Bangladesh (40.22%), while most participants had elementary school/associate degree in Pakistan (59%), Nepal (50.73%), and Cambodia (49.88%). With respect to household income, the proportion of participants in the lowest quintile (first) was higher than that of other groups except for participants in Cambodia, including the highest (fifth) most common. The proportion of urban residence was low in all six countries, ranging from 22.08% in Nepal to 46.71% in Indonesia.

Regarding women’s decision-making in daily life, Pakistan had the highest proportion of women who reported that their health care, household purchases, and visits to family or relatives were decided by the partner (47.31%, 48.28%, and 44.39%, respectively). Regarding women’s freedom from domination, the highest proportion of participants among six countries answered that their husband was justified in hitting or beating them if they went out without telling him in Pakistan (36.50%) and neglected the children in Cambodia (45.05%). Pakistan also had the highest proportion of women for all women’s self-assertion items that their husband was justified in hitting or beating them if they argued with husband (40.41%), refused to have sex (36.64%), and burned food (21.73%).

Among six countries, the country with the highest percentage of participants who did not use newspaper, radio, or television was Bangladesh (84.04%, 91.40%, and 41.75%, respectively), followed by Pakistan (76.96%, 84.29%, and 36.84%, respectively). The country with the highest percentage of participants who used mass media at least once a week was the Philippines (newspaper: 18.60%, radio: 46.89%, and television: 70.04%).

Among six countries, Pakistan had the lowest proportion of participants who reported that their children were vaccinated against BCG (77.56%), DPT (54.28%), and Measles (54.63%) while Indonesia had the lowest proportion of participants who reported that their children were vaccinated against Polio (69.31%).

### 3.2. Correlation Coefficient among SEP, Women Empowerment, and Media Use

Table 2 shows results of Pearson correlation coefficient analysis for examining the relationship among SEP, women empowerment (decision-making in daily life, freedom from domination, and self-assertion), and media use. A positive significant correlation existed between educational attainment and household income (r = 0.4416). Regarding women empowerment, there were positive significant correlations between sub-categories, with correlation coefficient ranging from 0.4010–0.6904. A weakly positive and significant correlation existed between media use type with correlation coefficient ranging from 0.1573–0.3141.

### 3.3. Women Decisional Authority and Vaccination for Children

Table 3 presents odds ratios (OR) and 95% confidence intervals (CI) of the dimensions of women’s decisional authority and media use associated with four types of vaccination. Women with higher decisional authority levels had significantly higher odds of getting their children vaccinated compared to women with lower decisional authority in Model I after controlling socio-demographic factors. Regarding decision-making, the likelihood of having their children vaccinated against BCG was higher among women who made decisions alone about her health care (OR = 1.31, 95% CI = 1.18–1.46), making major household purchases (OR = 1.16, 95% CI = 1.03–1.31), and visits to her family or relatives (OR = 1.14, 95% CI = 1.01–1.28) than those who did not have decision-making authority. Similar to results obtained after analyzing adoption of BCG vaccines, women who had high decisional authority levels were significantly more likely to get their children vaccinated against DPT, polio, and measles. Regarding their attitudes toward partner violence, participants who did not believe that a husband was justified in hitting his wife if she went out without telling him were more likely to get their children vaccinated against BCG (OR = 1.15, 95% CI = 1.04–1.27), polio (OR = 1.09, 95% CI = 1.01–1.17), and measles (OR = 1.08, 95% CI = 1.00–1.16) than were those who believed it was justified. Those who disagreed that a husband was justified in hitting or beating his wife if she argued with him were more likely to get their children vaccinated against BCG (OR = 1.19, 95% CI = 1.07–1.32) and measles (OR = 1.09, 95% CI = 1.01–1.17) than those who agreed that it was justified. However, the association between vaccination and attitudes toward partner violence if a woman neglected the children, refused to have sex, or burned food was not statistically significant.

When media use (newspaper, radio, and television) was added to Model II, the likelihood of having their children vaccinated (i.e., BCG, DPT, polio, and measles) was higher among those who frequently used media than those who did not use media at all after controlling for socio-demographic factors and women’s decisional authority. Participants who read a newspaper (OR = 2.80, 95% CI = 1.02–7.64) and watched television (OR = 1.86, 95% CI = 1.61–2.16) on a daily basis were more likely to get their children vaccinated against BCG than those who did not use either medium at all. Similarly, the likelihood of getting their children vaccinated against polio was higher among those who read newspaper at least once a week (OR = 1.13, 95% CI = 1.03–1.25) and listened to a radio daily (OR = 1.17, 95% CI = 1.08–1.34) than those who did not use either media at all. Regarding DPT, participants who frequently used media such as newspaper (OR = 1.21, 95% CI = 1.10–1.33), radio (OR = 1.25, 95% CI = 1.17–1.35), and television (OR = 1.86, 95% CI = 1.61–2.16) were more likely to have their children vaccinated against DPT than those who did not use media at all. Regarding measles vaccinations, it produced similar results. Participants who frequently used media such as newspaper (OR = 1.08, 95% CI = 1.01–1.14), radio (OR = 1.09, 95% CI = 1.02–1.17), and television (OR = 1.58, 95% CI = 1.42–1.77) were more likely to have their children vaccinated against measles than those who did not use media at all. In contrast, those who listened to radio on a daily basis (OR = 0.55, 95% CI = 0.35–0.86) were less likely to get their children vaccinated against BCG than those who did not listen to a radio at all. Also, the likelihood of getting their children vaccinated against DPT was lower among those who listened to a radio daily (OR = 0.62, 95% CI = 0.42–0.91) than those who did not listen to the radio at all.

## 4. Discussion

Our results showed that vaccination for children is significantly associated with women’s decisional authority and media use in South and Southeast Asia.

First, we found that women who had decision-making authority and were against domestic violence were more likely to have their children vaccinated. In the literature, physical abuse and domestic violence towards women are known to increase the risk of illness and death of newborn infants in developing countries of South and Southeast Asia [17,18]. Ultimately, enhancing health-related decisional authority and social status of women can act as a fundamental measure to improve maternal and child health status including vaccination [19]. Therefore, it is necessary to engage women in activities that extend rights of women and institutionalize gender equality throughout the society.

Second, media use was positively associated with vaccination of children. Newspaper and television use are known to be significantly associated with better child health such as vaccination [8,12,15,20]. This is consistent with other studies showing association between media use behaviors and mothers who have their children vaccinated [15]. This suggests that reducing communication inequalities to encourage mothers could also contribute to improvement in vaccine coverage for children in developing countries.

For East Asian mothers, decisional authority, self-efficacy, and health literacy can all increase the likelihood that they would vaccinate their children [21]. Vaccination for children can be encouraged by communication within families [22]. However, in Bangladesh and Pakistan, the important role played by mothers-in-laws has been found to be a barrier preventing their daughters-in-laws from utilizing preventive services including vaccination [23,24,25]. In most Asian countries including Nepal, mothers-in-laws make decisions for their daughters-in-laws, in accordance with patriarchal systems [25,26]. Women after marriage become husband’s family members and serve his parents. Although husbands can act as a support system for women to deal with their mothers-in-laws, the role of Asian men in childbirth is very limited [27,28]. Consequently, informing mothers-in-laws of the benefits of preventive healthcare services by providing them with health education is important for increase vaccine coverage rates of women in South and Southeast Asia [22,29,30,31].

Developing countries in South and Southeast Asia are undergoing significant changes. First, investments in improving women’s educational attainment are underway. Young educated women are more active in utilizing healthcare services as they have higher health literacy [23,32,33]. Second, efforts are being made to mitigate social inequality through economic growth. It is generally accepted that when gross national income per capita increases, life expectancy increases as well [34,35,36]. This is because unmet needs in health and medical services are satisfied when economy develops and household income increases [37,38,39]. However, current efforts to elevate women’s social status remain insufficient. When women are further empowered child health care indices will improve while infant mortality rates will decrease.

### Study Limitations

First, the present study used cross-sectional data to analyze only correlation, not causation. Although data collected used random effects model to explain heterogeneity among countries, it was impossible to control qualitative heterogeneity entirely among the six nations studied. This might have created biases when using the research model to estimate coefficients. While DHS has the advantage of conducting interstate analysis of data collected through standardized questionnaires and methods, it must be noted that, when interpreting results, investigations included in the present analysis were conducted over four years (2011–2014) and only representative populations in these developing countries were selected. Second, because women who participated in investigations responded regarding their past pregnancies/childbirths, there might be recall bias in responses. In addition, because the study used a secondary data, some measures were measured indirectly (e.g., decisions about child health). Third, potential confounding variables could also affect the statistical model of this study (e.g., fear of adverse reactions, vaccine availability, number of children/family size). Also, the prediction and use of main variables with various measurement items will increase the reliability and validity of the study in the future study. Finally, although decisions about vaccination may vary depending on the culture and traditions of each country (e.g., father’s role), this study did not incorporate these into the model.

## 5. Conclusions

The present study revealed that women’s decisional authority and the use of media were associated with improvements in vaccine coverage for children. Consequently, for child health care in developing countries in South and Southeast Asia, measures that can assist families and local communities in promoting women’s health decisional authority must be contemplated. Empowered women can give birth to healthy fetuses through utilization of appropriate child healthcare, leading to a population that can reproduce more healthily. This can ultimately improve economic and human capital of developing countries.

## Figures and Tables

**Table 1:** General characteristics of the study sample (N = 45,168).

		Bangladesh (2011) N = 6917		Cambodia (2014) N = 6169		Indonesia (2012) N = 15579		Nepal (2014) N = 3369		Pakistan (2012) N = 7009		Philippines (2013) N = 6125	
		N	(%)	N	(%)	N	(%)	N	(%)	N	(%)	N	(%)
Age													
	13–19	791	11.44	187	3.03	473	3.04	132	3.92	111	1.58	181	2.96
	20–24	2374	34.32	1423	23.07	2865	18.39	848	25.17	1049	14.97	1269	20.72
	25–29	1966	28.42	1975	32.01	4476	28.73	1194	35.44	2063	29.43	1547	25.26
	30–34	1105	15.98	1635	26.50	3882	24.92	653	19.38	1951	27.84	1407	22.97
	35–39	472	6.82	613	9.94	2601	16.70	349	10.36	1209	17.25	1036	16.91
	40–44	178	2.57	258	4.18	1079	6.93	149	4.42	486	6.93	510	8.33
	45–49	31	0.45	78	1.26	203	1.30	44	1.31	140	2.00	175	2.86
Educational attainment													
	Uneducated	1406	20.33	813	13.18	399	2.56	1709	50.73	4135	59.00	127	2.07
	Elementary school/associate degree	2158	31.20	3077	49.88	4616	29.63	642	19.06	941	13.43	1436	23.44
	Middle school/associate degree	2782	40.22	2037	33.02	8357	53.64	827	24.55	1119	15.97	2991	48.83
	High school/associate degree or higher	571	8.26	242	3.92	2207	14.17	191	5.67	814	11.61	1571	25.65
Household income													
	First quintile (the lowest)	1650	23.85	1401	22.71	4442	28.51	1187	35.23	1787	25.50	2010	32.82
	Second quintile	1395	20.17	1169	18.95	3119	20.02	649	19.26	1476	21.06	1415	23.10
	Third quintile	1278	18.48	964	15.63	2847	18.27	519	15.41	1314	18.75	1133	18.50
	Fourth quintile	1272	18.39	1079	17.49	2696	17.31	479	14.22	1239	17.68	897	14.64
	Fifth quintile (the highest)	1322	19.11	1556	25.22	2475	15.89	535	15.88	1193	17.02	670	10.94
Location													
	Urban	2187	31.62	1705	27.64	7277	46.71	744	22.08	3006	42.89	2409	39.33
	Rural	4730	68.38	4464	72.36	8302	53.29	2625	77.92	4003	57.11	3716	60.67
Decision-making in daily life													
(1) Woman’s health care													
	Partner decides	2317	33.50	496	8.04	2329	14.95	992	29.44	3316	47.31	259	4.23
	Jointly	3819	55.21	3079	49.91	7776	49.91	1540	45.71	2999	42.79	2815	45.96
	Woman decides alone	781	11.29	2594	42.05	5474	35.14	837	24.84	694	9.90	3051	49.81
(2) Household purchases													
	Partner decides	2463	35.61	355	5.75	2646	16.98	1094	32.47	3384	48.28	793	12.95
	Jointly	4037	58.36	4946	80.18	9857	63.27	1052	31.23	3152	44.97	4099	66.92
	Woman decides alone	417	6.03	868	14.07	3076	19.74	1223	36.30	473	6.75	1233	20.13
(3) Visiting to family or relatives													
	Partner decides	2338	33.80	196	3.18	2321	14.90	873	25.91	3111	44.39	399	6.51
	Jointly	3997	57.79	4838	78.42	11,248	72.20	1452	43.10	3300	47.08	4261	69.57
	Woman decides alone	582	8.41	1135	18.40	2010	12.90	1044	30.99	598	8.53	1465	23.92
Freedom from domination													
(1) Goes out without telling him													
	Yes	1170	16.91	2084	33.78	4257	27.33	10	0.30	2558	36.50	341	5.57
	No	5747	83.09	4085	66.22	11,322	72.67	3359	99.70	4451	63.50	5784	94.43
(2) Neglects the children													
	Yes	1351	19.53	2779	45.05	4603	29.55	14	0.42	2619	37.37	801	13.08
	No	5566	80.47	3390	54.95	10,976	70.45	3355	99.58	4390	62.63	5324	86.92
Self-assertion													
(1) Argues with him													
	Yes	1606	23.22	1533	24.85	1191	7.64	7	0.21	2832	40.41	242	3.95
	No	5311	76.78	4636	75.15	14,388	92.36	3362	99.79	4177	59.59	5883	96.05
(2) Refuses to have sex													
	Yes	611	8.83	758	12.29	1493	9.58	4	0.12	2568	36.64	130	2.12
	No	6306	91.17	5411	87.71	14,086	90.42	3365	99.88	4441	63.36	5995	97.88
(3) Burns food													
	Yes	281	4.06	704	11.41	516	3.31	2	0.06	1523	21.73	145	2.37
	No	6636	95.94	5465	88.59	15,063	96.69	3367	99.94	5486	78.27	5980	97.63
Newspaper use													
	Not at all	5813	84.04	4745	76.92	7879	50.57	2560	75.99	5394	76.96	2685	43.84
	Less than once a week	679	9.82	1071	17.36	5672	36.41	535	15.88	1321	18.85	2301	37.57
	At least once a week	425	6.14	353	5.72	2028	13.02	274	8.13	113	1.61	1139	18.60
	Daily	0	0.00	0	0.00	0	0.00	0	0.00	181	2.58	0	0.00
Radio use													
	Not at all	6322	91.40	3064	49.67	7929	50.90	796	23.63	5908	84.29	1357	22.16
	Less than once a week	293	4.24	1572	25.48	5267	33.81	1303	38.68	904	12.90	1896	30.96
	At least once a week	302	4.37	1533	24.85	2383	15.30	1270	37.70	58	0.83	2872	46.89
	Daily	0	0.00	0	0.00	0	0.00	0	0.00	139	1.98	0	0.00
Television use													
	Not at all	2888	41.75	1871	30.33	914	5.87	1310	38.88	2582	36.84	769	12.56
	Less than once a week	853	12.33	947	15.35	1817	11.66	930	27.60	1313	18.73	1066	17.40
	At least once a week	3176	45.92	3351	54.32	12,848	82.47	1129	33.51	148	2.11	4290	70.04
	Daily	0	0.00	0	0.00	0	0.00	0	0.00	2966	42.32	0	0.00
Received BCG													
	No	395	5.71	385	6.24	2438	15.65	272	8.07	1573	22.44	482	7.87
	Yes	6522	94.29	5784	93.76	13,141	84.35	3097	91.93	5436	77.56	5643	92.13
Received DPT													
	No	1029	14.88	1276	20.68	5659	36.32	548	16.27	3174	45.28	1420	23.18
	Yes	5888	85.12	4893	79.32	9920	63.68	2821	83.73	3835	54.72	4705	76.82
Received Polio													
	No	1015	14.67	1296	21.01	4781	30.69	533	15.82	1810	25.82	1517	24.77
	Yes	5902	85.33	4873	78.99	10,798	69.31	2836	84.18	5199	74.18	4608	75.23
Received Measles													
	No	1665	24.07	1721	27.90	4949	31.77	821	24.37	3198	45.63	1719	28.07
	Yes	5252	75.93	4448	72.10	10,630	68.23	2548	75.63	3811	54.37	4406	71.93

**Table 2:** Correlation coefficient among SEP, women empowerment, and media use.

	Educational Attainment	Household Income	Woman’s Health Care	Household Purchases	Visiting to Family or Relatives	Goes out without Telling him	Neglects the Children	Argues with him	Refuses to Have Sex	Burns Food	Newspaper Use	Radio Use	Television Use
Educational attainment	1												
Household income	0.442	1											
Woman’s health care	0.213	0.055	1										
Household purchases	0.164	0.053	0.470	1									
Visiting to family or relatives	0.154	0.055	0.465	0.517	1								
Goes out without telling him	0.162	0.134	0.064	0.061	0.086	1							
Neglects the children	0.133	0.122	0.040	0.037	0.054	0.690	1						
Argues with him	0.253	0.132	0.140	0.134	0.139	0.514	0.523	1					
Refuses to have sex	0.223	0.128	0.146	0.122	0.129	0.477	0.464	0.560	1				
Burns food	0.202	0.127	0.105	0.097	0.100	0.401	0.403	0.500	0.537	1			
Newspaper use	0.503	0.351	0.143	0.113	0.092	0.104	0.094	0.171	0.121	0.122	1		
Radio use	0.212	0.089	0.166	0.129	0.141	0.098	0.079	0.153	0.125	0.097	0.314	1	
Television use	0.400	0.436	0.112	0.095	0.072	0.080	0.069	0.167	0.107	0.129	0.314	0.157	1

The significance value of the association between all variables was less than 0.001.

**Table 3:** Odds ratio (OR) and 95% confidence interval (CI) of women empowerment and media use with four types of vaccination among six South and Southeast Asian countries in the DHS data (N = 45,168).

		BCG				DPT				Polio				Measles			
		Model I		Model II		Model I		Model II		Model I		Model II		Model I		Model II	
		aOR	95% CI	aOR	95% CI	aOR	95% CI	aOR	95% CI	aOR	95% CI	aOR	95% CI	aOR	95% CI	aOR	95% CI
Decision-making (1) woman’s health care																	
	Partner decides (Ref.)																
	Jointly	1.18 ^†^	1.07, 1.30	1.17 ^†^	1.07, 1.30	1.12 ^†^	1.04, 1.20	1.12 ^†^	1.04, 1.20	1.01	0.94, 1.09	1.01	0.94, 1.09	1.06	0.99, 1.14	1.05	0.98, 1.13
	Woman decides alone	1.31 *	1.18, 1.46	1.30 *	1.18, 1.45	1.23 *	1.14, 1.33	1.22 *	1.13, 1.32	1.11 +	1.03, 1.21	1.11 +	1.02, 1.20	1.16 *	1.08, 1.25	1.15 *	1.07, 1.24
Decision-making (2) household purchases																	
	Partner decides (Ref.)																
	Jointly	1.19 ^†^	1.08, 1.32	1.17 ^†^	1.06, 1.29	1.14 ^†^	1.06, 1.23	1.13 ^†^	1.05, 1.22	1.13 ^†^	1.05, 1.22	1.12 ^†^	1.04, 1.21	1.12 ^†^	1.05, 1.20	1.11 ^†^	1.03, 1.19
	Woman decides alone	1.16 +	1.03, 1.31	1.14 +	1.01, 1.28	1.09	1.00, 1.19	1.08	0.99, 1.18	1.09	1.00, 1.19	1.09	1.00, 1.19	1.12 +	1.03, 1.21	1.10 +	1.02, 1.20
Decision-making (3) visiting to family or relatives																	
	Partner decides (Ref.)																
	Jointly	1.28 *	1.16, 1.42	1.29 *	1.16, 1.42	1.28 *	1.18, 1.38	1.28 *	1.19, 1.38	1.23 *	1.14, 1.33	1.23 *	1.14, 1.33	1.15 *	1.07, 1.24	1.15 *	1.07, 1.24
	Woman decides alone	1.14 +	1.01, 1.28	1.16 +	1.03, 1.31	1.18 *	1.08, 1.29	1.19 *	1.08, 1.30	1.03	0.94, 1.13	1.05	0.95, 1.15	1.12 ^†^	1.02, 1.22	1.12 ^†^	1.03, 1.22
Attitudes on partner violence (1) goes out without telling him																	
	Yes (Ref.)																
	No	1.15 ^†^	1.04, 1.27	1.15 ^†^	1.04, 1.27	1.07	0.99, 1.15	1.07	0.99, 1.15	1.09 +	1.01, 1.17	1.10 +	1.02, 1.18	1.08 +	1.00, 1.16	1.08 +	1.00, 1.16
Attitudes on partner violence (2) neglects the children																	
	Yes (Ref.)																
	No	0.94	0.85, 1.03	0.95	0.86, 1.05	0.99	0.92, 1.06	0.99	0.93, 1.07	0.97	0.90,1.04	0.97	0.91, 1.05	0.96	0.89, 1.02	0.96	0.90, 1.03
Attitudes on partner violence (3) argues with him																	
	Yes (Ref.)																
	No	1.19 +	1.07, 1.32	1.15 +	1.03, 1.28	1.08 +	1.00, 1.17	1.07	0.98, 1.16	1.08	0.99, 1.17	1.06	0.97, 1.15	1.09 +	1.01, 1.17	1.07	0.99, 1.15
Attitudes on partner violence (4) refuses to have sex																	
	Yes (Ref.)																
	No	0.98	0.88, 1.09	0.98	0.88, 1.10	1.07	0.98, 1.16	1.07	0.98, 1.17	0.99	0.91, 1.08	1.00	0.92, 1.09	1.00	0.92, 1.08	0.99	0.91, 1.08
Attitudes on partner violence (5) burns food																	
	Yes (Ref.)																
	No	1.08	0.95, 1.22	1.04	0.92, 1.18	1.02	0.93, 1.13	1.00	0.90, 1.11	0.98	0.88, 1.09	0.96	0.87, 1.07	1.02	0.93, 1.13	1.00	0.91, 1.10
Newspaper use																	
	Not at all (Ref.)																
	Less than once a week			1.15 ^†^	1.06, 1.26			1.09 ^†^	1.03, 1.16			1.06	0.99, 1.12			1.08 +	1.01, 1.14
	At least once a week			1.27 ^†^	1.08, 1.48			1.21 *	1.10, 1.33			1.13 +	1.03, 1.25			1.09 +	1.00, 1.19
	Daily			2.80 +	1.02, 7.64			1.36	0.92, 2.01			0.90	0.59, 1.36			1.43	0.99, 2.07
Radio use																	
	Not at all (Ref.)																
	Less than once a week			1.15 ^†^	1.06, 1.25			1.08 +	1.02, 1.14			1.10 ^†^	1.04, 1.17			1.02	0.97, 1.08
	At least once a week			1.36 *	1.23, 1.52			1.25 *	1.17, 1.35			1.25 *	1.16, 1.34			1.09 ^†^	1.02, 1.17
	Daily			0.55 ^†^	0.35, 0.86			0.74	0.52, 1.08			0.62 +	0.42, 0.91			0.80	0.56, 1.14
Television use																	
	Not at all (Ref.)																
	Less than once a week			1.53 *	1.39, 1.69			1.24 *	1.15, 1.34			1.33 *	1.24, 1.44			1.29 *	1.20, 1.39
	At least once a week			1.78 *	1.61, 1.96			1.37 *	1.28, 1.48			1.57 *	1.46, 1.69			1.34 *	1.25, 1.43
	Daily			1.86 *	1.61, 2.16			1.36 *	1.22, 1.53			1.17 ^†^	1.08, 1.34			1.58 *	1.42, 1.77

BCG = bacille de Calmette-Guerin vaccine, DPT = diphtheria, pertussis, and tetanus vaccine, a = adjusted age, educational attainment, household income, location. ^*^ = *p*-value < 0.001, ^†^ = *p*-value < 0.01, ^+^ = *p*-value < 0.05 indicates statistical significance.
